# Genome-wide association analysis identified both RNA-seq and DNA variants associated to paratuberculosis in Canadian Holstein cattle ‘in vitro’ experimentally infected macrophages

**DOI:** 10.1186/s12864-021-07487-4

**Published:** 2021-03-07

**Authors:** Olivier Ariel, Jean-Simon Brouard, Andrew Marete, Filippo Miglior, Eveline Ibeagha-Awemu, Nathalie Bissonnette

**Affiliations:** 1 Sherbrooke Research and Development Centre, Agriculture and Agri-Food Canada, Sherbrooke, QC J1M 0C8 Canada; 2 Center of Genetic Improvement of Livestock, University of Guelph, Guelph, ON N1G 2W1 Canada; 3 Canadian Dairy Network, Guelph, ON N1K 1E5 Canada

**Keywords:** *Mycobacterium avium* subspecies *paratuberculosis*, Dairy bovine, Macrophage, RNA-sequencing, Genotyping, Genome-wide association studies, SNP

## Abstract

**Background:**

*Mycobacterium avium ssp. paratuberculosis* (MAP) is the causative agent of paratuberculosis, or Johne’s disease (JD), an incurable bovine disease. The evidence for susceptibility to MAP disease points to multiple interacting factors, including the genetic predisposition to a dysregulation of the immune system. The endemic situation in cattle populations can be in part explained by a genetic susceptibility to MAP infection. In order to identify the best genetic improvement strategy that will lead to a significant reduction of JD in the population, we need to understand the link between genetic variability and the biological systems that MAP targets in its assault to dominate macrophages. MAP survives in macrophages where it disseminates. We used next-generation RNA (RNA-Seq) sequencing to study of the transcriptome in response to MAP infection of the macrophages from cows that have been naturally infected and identified as positive for JD (JD (+); *n* = 22) or negative for JD (healthy/resistant, JD (−); *n* = 28). In addition to identifying genetic variants from RNA-seq data, SNP variants were also identified using the Bovine SNP50 DNA chip.

**Results:**

The complementary strategy allowed the identification of 1,356,248 genetic variants, including 814,168 RNA-seq and 591,220 DNA chip variants. Annotation using SnpEff predicted that the 2435 RNA-seq genetic variants would produce high functional effect on known genes in comparison to the 33 DNA chip variants. Significant variants from JD(+/−) macrophages were identified by genome-wide association study and revealed two quantitative traits loci: BTA4 and 11 at (*P* < 5 × 10^− 7^). Using BovineMine, gene expression levels together with significant genomic variants revealed pathways that potentially influence JD susceptibility, notably the energy-dependent regulation of mTOR by LKB1-AMPK and the metabolism of lipids.

**Conclusion:**

In the present study, we succeeded in identifying genetic variants in regulatory pathways of the macrophages that may affect the susceptibility of cows that are healthy/resistant to MAP infection. RNA-seq provides an unprecedented opportunity to investigate gene expression and to link the genetic variations to biological pathways that MAP normally manipulate during the process of killing macrophages. A strategy incorporating functional markers into genetic selection may have a considerable impact in improving resistance to an incurable disease. Integrating the findings of this research into the conventional genetic selection program may allow faster and more lasting improvement in resistance to bovine paratuberculosis in dairy cattle.

**Supplementary Information:**

The online version contains supplementary material available at 10.1186/s12864-021-07487-4.

## Background

*Mycobacterium avium* subspecies *paratuberculosis* (MAP) is a causative agent for paratuberculosis or Johne’s disease (JD). Bovine paratuberculosis has been a global concern for many years. It inflicts a substantial economic burden on dairy and beef industries worldwide [1–3]. The prevalence of infected farms has been increasing worldwide, reaching 66% for farms in Western Canada [4], 68% in USA [5, 6] and 68% in Great Britain [7]. Paratuberculosis induces substantial economic losses ranging from early culling of JD cows to decreased milk production and lowered reproductive and feed efficiency [8].

Paratuberculosis is a slow and progressive chronic inflammatory bowel disease, leading to malfunction of the intestinal tract and persistent diarrhea [1, 3, 9]. These symptoms, coupled with serological tests (e.g., ELISA) and MAP detecting assays (e.g., fecal PCR), allow detecting clinical JD [3, 10]. With cows confirmed JD (+) by bacterial culture, the ability to detect actual MAP infections using ELISA is 96–99% [11]. However, for cows excreting a low amount of MAP in feces, ELISA assay’s sensitivity can be low as 4.8% [12] indicating a lack sensitivity for detecting subclinical JD probably because the subclinical cows shed MAP intermittently in their feces [13–15]. Besides, the culture of MAP is labor-intensive and often compromised by bacterial contamination [16]. In contrast, PCR’s fecal analysis is cost-effective, rapid and compensates for the weak sensitivity of culture for diagnosis [17–20]. Though direct fecal PCR outperformed ELISA in detecting cows excreting MAP in feces [12, 17], a better MAP testing strategy would be concurrent serological and fecal testing with the repeated diagnosis over time. Such a two-fold strategy would improve sensitivity because animals with subclinical diseases may shed MAP intermittently in their feces [17, 21].

Though routine farm management practices such as test and cull have shown limited performance, several modeling studies have demonstrated that vaccination is an economically attractive option for dairy producers [22, 23], resulting in benefits such as delay in the onset of clinical disease, reduced clinical cases, and reduced level of MAP shedding [24–26]. However, vaccination does not prevent new infection and must be administered to each new individual in each generation. The results of genetic improvement of disease resistance are permanent; genetic gains made in one generation remain in future generations, and under a program of continuous improvement, advances in genetic resistance accumulate generation upon generation [27]. In an ideal situation, vaccination should be combined with genetic improvement as both will contribute to eradicating the incurable disease. Furthermore, host genetic improvement of JD resistance is a good strategy for reducing new infections, but improvement is a slow, long-term process [28]. For bovine tuberculosis, the model predicted that the risk would be reduced by half after 4, 6, 9, and 15 generations for selection intensities corresponding to genetic selection of the 10, 25, 50, and 70% most resistant sires, respectively [29]. To develop a genetic improvement strategy that will lead to a significant reduction of JD in the population, a better understanding of the genetic components influencing the biological systems that MAP targets during its assault is required. Early events of MAP infection occur in two functional stages: (1) Invasion through the intestinal barrier via MAP discharge from epithelial M cells (2) phagocytosis and survival in macrophages [30–32]. It is known that MAP uses tissue resident macrophages as its primary reservoir for survival and for multiplication [33–35]. On the one hand, studies of the effects of age and dose on MAP infection susceptibility in experimental infection models and naturally infected calves reported significant individual variation, which could be explained by the host’s genetic difference in susceptibility/resistance to MAP disease [36, 37]. On the other hand, several genetic variations were associated with the susceptibility to develop clinical JD [38–44], notably in the macrophage *BOLA-DRB2* gene [44]. Interestingly, genetic variations in numerous candidate genes expressed in macrophages are associated with resistance/susceptibility to MAP infection, notably the *NOD2* [45, 46], *IL10* [47–50], *SLC11A1*, and Toll-like receptor genes [51, 52]. Genetic variations in *NOD2* associated with Crohn’s disease can predict impaired innate immunity [53, 54]. In JD ruminants this MAP-induced granulomatous infection shares many features with Crohn’s disease [55, 56] and has similarities with ileocecal tuberculosis [57].

Nowadays, sequencing technology is becoming more and more affordable and the accuracy of the SNPs called from the RNA-seq data, compared to whole-genome sequencing, is > 98% [58]. Augmenting association studies with RNA sequencing (RNA-seq) can detect subtle pathways that might be affected by genetic variations since the design of RNA-seq allows one to read the genome activity of a cell or a system in a defined environment and at given time points. In a previous study, we have defined the transcriptomic profiles of bovine macrophages from naturally MAP infected cows, i.e., JD(+) cows, and otherwise healthy cows, i.e., JD(−) [59]. We analyzed the phenotypic response to MAP infection and identified differentially expressed genes associated with inflammatory processes, the resolution of inflammation, and cellular metabolism, among others. Interestingly, the transcriptomic profiles of JD(+) macrophages differed distinctly from JD(−) macrophages. In the current study, we investigate the potential of an in vitro model of macrophages to identify individual genetic variations from the transcriptome associated with bovine paratuberculosis. We hypothesize that the genetic variations identified in the transcriptome associated with disease susceptibility could provide information on (1) the biological pathways leading to susceptibility to MAP infection’s susceptibility and (2) the genetic markers providing weakness to the host at the early stage of MAP infection.

This research’s originality comes from using two datasets: (1) SNP variants mined from differentially expressed in vitro infected macrophages from both healthy and JD cows, and (2) DNA chip data to provide high-resolution genomic analysis of macrophages. Taken together, the result of this functional genomics study provides useful information for predicting the genotype-to-phenotype relationship in this context of host-pathogen interaction where macrophages are targeted by MAP to survive and to escape the normal mycobacterial killing process.

## Results

### RNA-Seq variants and DNA-derived genotypes

**Fig. 1:**
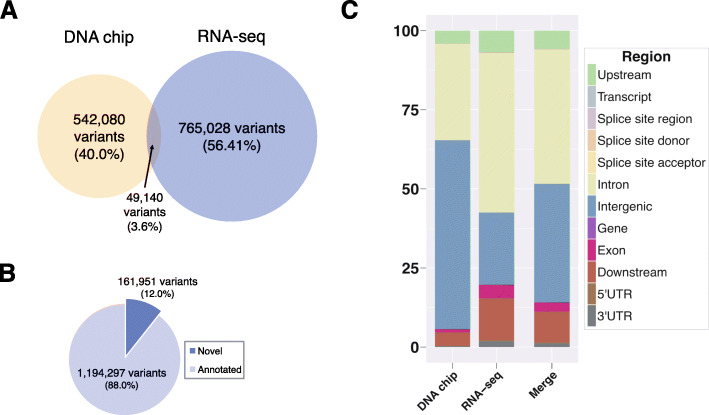
Functional characterization of all variants identified using DNA chips and RNA-seq. SNPs were identified from the bovine genome while RNA-seq variants were identified from the transcriptome of bovine macrophages. The distribution of variants by the different methods used to detect them is represented in (**a**) and the proportion of novel and annotated variants is represented in (**b**). The genomic distributions of variants from each methods is represented in (**c**)

The objective of our work was to use the genetic information from the transcripts and regulatory sequences of the macrophages from JD(−) and 22 JD(+) cows in the genetic association study. Genetic variants from the resting macrophages (unchallenged controls) and those called to respond to MAP infection were combined with the imputed SNPs to obtain a dense portray of the genetics variations of these innate immune sentinel cells. For each cow, RNA-seq samples from all infection time points were merged with the RNA-seq sample of the unchallenged controls. For each cow, the pool is a representation of all the genes expressed in the macrophages at one point or another during infection, including genes expressed in the resting unchallenged macrophages. The in vitro infection model makes it possible to include genes expressed in response to a MAP infection for the search for genetic variations. Overall, the transcriptome of the macrophages from 28 JD(−) and 22 JD(+) cows was sequenced, resulting in 9820 billion paired-end reads. To avoid false calls, only unique mapping reads were used and these included 473.3 ± 33.0 million mapped unique reads per cow for the first 12 cows (previous study [59]) and 84.26 ± 34.02 per cow for the 38 additional cows. After filtering to generate a non-redundant dataset (Supplementary Figure 1), we identified 1,356,248 variants, including 814,168 RNA-seq, and 591,220 DNA chip variants (Table 1). Of these 1,356,248 variants, 104,065 were insertions or deletions (indels). Comparing matched RNA and DNA sequences enables an assessment of the accuracy of SNP RNA calls. Correlation of genotype called between the common SNPs of RNA-seq and SNP50 was of 98.8 ± 0.7%. The RNA-seq data alone enabled discovery of 765,028 (56.4%) of all the genetic variants identified, while the imputed DNA chips represent 40% (542,060) of the identified SNPs (Fig. 1a). A total of 88% of the variants (Fig. 1b) were found annotated in dbSNP (version 150).

**Table 1:** Summary statistics of the identified variants using the respective methods, from the bovine genome using DNA chip and from the transcriptome of the macrophages using RNA-seq

Genotyping methods (counts)	DNA chip	RNAseq	Merge
Variants processed			
SNPs	591,220	814,168	1,356,248
Insertions	0	50,272	50,272
Deletions	0	43,007	43,007
Categories			
MISSENSE	2203	17,644	18,788
NONSENSE	4	96	99
SILENT	4473	25,954	27,879
Effects by impact			
HIGH	33	2435	2459
LOW	5109	28,359	30,762
MODERATE	2202	17,996	19,139
MODIFIER	664.774	1,053,336	1,655,791
Effects by type and region			
3_prime_UTR_variant	2110	19,914	20,494
5_prime_UTR_premature_start_codon	58	459	483
5_prime_UTR_truncation	0	1	1
5_prime_UTR_variant	318	3506	3648
bidirectional_gene_fusion	0	1	1
conservative_inframe_deletion	0	103	103
conservative_inframe_insertion	0	77	77
disruptive_inframe_deletion	0	137	137
disruptive_inframe_insertion	0	90	90
downstream_gene_variant	28,694	146,242	166,616
exon_loss_variant	0	1	1
frameshift_variant	0	1754	1754
gene_fusion	0	1	1
initiator_codon_variant	0	2	2
intergenic_region	401,382	251,262	640,031
intragenic_variant	0	2	2
intron_variant	205,303	557,864	727,911
missense_variant	2202	17,628	18,771
non_coding_transcript_exon_variant	200	1611	1781
non_coding_transcript_variant	0	10	10
splice_acceptor_variant	12	496	504
splice_donor_variant	16	590	602
splice_region_variant	735	3146	3704
start_lost	1	12	13
stop_gained	4	118	121
stop_lost	0	12	12
stop_retained_variant	2	18	18
synonymous_variant	4471	25,936	27,861
upstream_gene_variant	27,358	75,567	98,406

### Enrichment of genetic variants in functional categories

We annotated and predicted the variants’ effects using SnpEff. Table 1 summarizes the regulatory functions of the effect (high, low, or moderate). As expected, due to the nature of the sequencing method, RNA-seq dataset had the most SNP having a high functional impact. The SNPs identified from RNA-seq data are enriched in the expressed gene regions. This enrichment is advantageous compared to DNA genotyping methods because it increases the power to detect the SNPs responsible for regulating gene expression. A higher proportion of intergenic SNPs was found with the DNA chips, while intronic, exonic, and UTRs variants were primarily identified in the RNA-seq dataset (Fig. 1c).

### Genome wide association study (GWAS) and pathway analysis of the significant variants

**Fig. 2:**
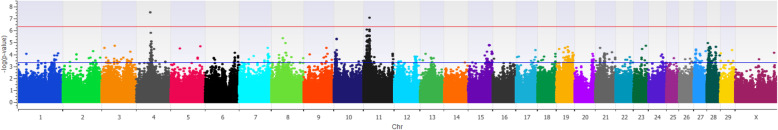
Manhattan plot of SNPs associated with Johne’s disease infection in dairy bovine. The –log_10_ of the *P*-value of variants are plotted. The red line represents a p-value of 5 × 10^−7^ while the blue line represent a *p*-value of 5 × 10^−4^

**Fig. 3:**
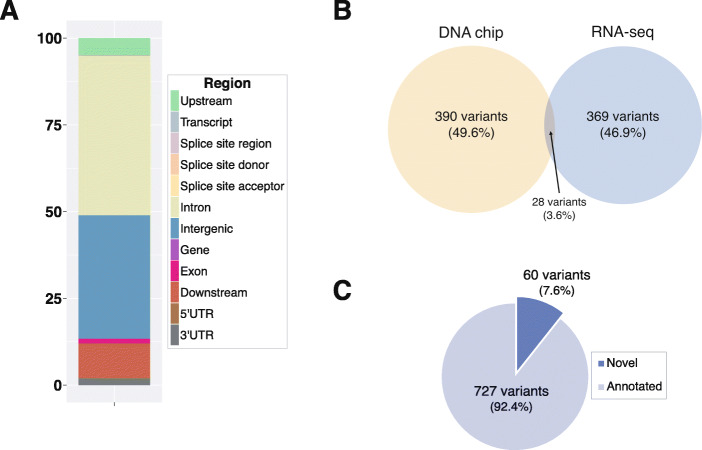
Functional characterization of variants significantly associated with JD in dairy bovine in the GWAS analysis at a *p*-value≤0.005. The distribution of the significant variants on the genome (**a**) and by the different method of sequencing used to detect them (**b**). The novel and annotated variants are represented in (**c**)

The main single cluster in the PCA plot (Supplementary Figure 2) and the deviation from the diagonal at the upper-right end of the Q-Q plot (Supplementary Figure 3) indicate absence of population stratification or other problems with the data, such as cryptic relatedness. Even though the pedigree information is known, inferring relationships through genomic marker data validates the absence of closely-related animals. The 1,356,248 SNPs called from the 50 cows, which includes the imputed RNA-sequencing and DNA chip datasets from the 28 JD(−) and 22 JD(+) cows, were used for performing the genome-wide association analysis (Fig. 2). A total of 787 variants (*P* ≤ 5 × 10^− 4^) were identified as significant (Table 2). Nine SNPs are within a distance of 1 Kb of the transcription start site (data not shown). Numerous eQTL were identified, mainly on BTA 4, 6, 7, 8, 10, 11, 12, 15, 27, and BTA28 (Fig. 2, blue line). The genomic distribution of these 787 variants (*P* ≤ 5 × 10^− 4^) suggests a higher enrichment in intronic compared to exonic sequences (Fig. 3a).

**Table 2:** Number of variants identified using the respective genotyping methods, from the bovine genome using DNA chip and from the transcriptome of the macrophages using RNA-seq

Genotyping Method	Total variants ^a^	Significant^b^ variants identified by GWAS				
		*p* ≤ 5 × 10^− 4^	*p* ≤ 5 × 10^−5^	*p* ≤ 5 × 10^− 6^	*p* ≤ 5 × 10^− 7^	*p* ≤ 5 × 10^− 8^
DNA chip	46,891					
RNA-seq	763,723					
Merge (with indel)	1,356,248	787	143	23	3	2

^a^ Post-filtration considering call rate, MAF = 5e-3, HWE = 1e-4, genotype quality, and read depth

^b^All variants were corrected at FDR = 0.05. For low MAF variants a suggestive threshold of 5e-4 was used

The 20 most significant variants are shown in Table 3. Only three variants passed the threshold of strong association (*P* < 1 × 10^− 7^). The two most significant SNPs were identified on BTA4 position 50 Mb (*P* = 3.26 × 10^− 8^). With an additional variant located on BTA11 (21.4 Mb; *P* = 9.25 × 10^− 8^) and two also on BTA11 (21.5 Mb, *P* = 1.26 × 10^− 8^; 12.4 Mb, *P* = 9.20 × 10^− 7^), genetic variants on BTA4 and 11 show a high association with JD-positive status. Considering the 143 SNP below the threshold of *P* < 5 × 10^− 5^, 15 and 82 additional SNP strengthened the association with BTA4 and BTA11, respectively. At this level of significance, the number of variants identified using the DNA chip (390 variants) and RNA-seq are similar. Only 28 variants were identified by both methods (Fig. 3b). In the 787 significant variants at *P* ≤ 5 × 10^− 4^, a total of 60 were not identified in the NCBI database, while the others (727) were already known (Fig. 3c).

**Table 3:** Top 20 most significant variants in the GWAS analysis

rsID	Chrom:Position	Ref/Alt alleles	***p***-value^a^	Gene	Ensembl ID	Impact
rs109231144	4:49533097	C/T	3.26e-08	*NRCAM*	ENSBTAG00000006732	MODIFIER
rs41650658	4:49535036	A/G	3.26e-08	*NRCAM*	ENSBTAG00000006732	MODIFIER
rs43668743	11:21401262	T/G	9.25e-08	*SOS1*	ENSBTAG00000011643	MODIFIER
rs43667381	11:21438609	T/A	8.89e-07	ENSBTAG00000037586	ENSBTAG00000037586	MODIFIER
rs43664819	11:12392739	T/C	9.20e-07	*CYP26B1, U6*	ENSBTAG00000012212	MODIFIER
rs383441105	11:21591340	G/A	1.26e-06	*U6*	ENSBTAG00000043391	MODIFIER
rs43387851	4:50001287	G/T	1.67e-06	*NME8, SFRP4*	ENSBTAG00000015353	MODIFIER
rs43668789	11:21312462	G/A	2.66e-06	*ARHGEF33*	ENSBTAG00000017039	MODIFIER
rs137226813	11:13989555	C/T	2.67e-06	*TGFA*	ENSBTAG00000000783	MODIFIER
rs134348861	11:15327632	A/G	2.70e-06	*TTC27*	ENSBTAG00000033010	MODIFIER
rs42051552	11:15339987	A/G	2.70e-06	*TTC27*	ENSBTAG00000033010	MODIFIER
rs43667373	11:21436273	G/A	3.20e-06	ENSBTAG00000037586	ENSBTAG00000037586	MODIFIER
rs379213820	11:21398019	C/G	3.20e-06	*SOS1*	ENSBTAG00000011643	MODIFIER
rs43666834	11:12281788	T/C	3.22e-06	*EXOC6B*	ENSBTAG00000020799	MODIFIER
rs41659204	11:12299398	T/C	3.22e-06	*EXOC6B*	ENSBTAG00000020799	MODIFIER
rs43665805	11:12321351	A/C	3.22e-06	*EXOC6B*	ENSBTAG00000020799	MODIFIER
rs43665815	11:12332275	A/G	3.22e-06	*EXOC6B*	ENSBTAG00000020799	MODIFIER
rs43665822	11:12338121	C/T	3.22e06	*EXOC6B*	ENSBTAG00000020799	MODIFIER
rs43667796	11:12415887	G/A	3.22e-06	*CYP26B1, U6*	ENSBTAG00000012212	MODIFIER
rs43663907	11:12434753	A/G	3.22e-06	*CYP26B1*, *U6*	ENSBTAG00000012212	MODIFIER

^a^All variants were corrected at FDR = 0.05. For low MAF variants a suggestive threshold of 5e-4 was used

GWAS, however, empowered significant molecular biomarker identification, enabling the association of pathways in the context of JD resistance/susceptibility. Using BovineMine, analysis revealed interesting pathways that deserved investigation (Table 4). Among the top 15 pathways being significantly enriched by variants with a *p*-value ≤0.005, were identified the metabolism of vitamins, lipids, and endogenous sterols. Many pathway associated with different aspects of cell metabolism are significantly enriched. In addition, four are not related to amino acid/lipids metabolism or cellular energy production. These pathways are implicated in DNA repairs and cell signaling.

**Table 4:** Top 15 pathways significantly enriched by genes associated with SNPs significant (−log[*p*-value] ≥3) in GWAS ^a^ performed using the merged dataset of genotypes (DNA and RNA-seq)

Pathways	Reactome term	***p***-value
Metabolism of water-soluble vitamins and cofactors	R-BTA-196849	0.0002
Endogenous sterols	R-BTA-211976	0.0005
Metabolism of vitamins and cofactors	R-BTA-196854	0.0019
Regulation of pyruvate dehydrogenase (PDH) complex	R-BTA-204174	0.0024
Metabolism	R-BTA-1430728	0.0047
Nicotinate metabolism	R-BTA-196807	0.0051
Pyruvate metabolism and Citric Acid (TCA) cycle	R-BTA-71406	0.0060
Signaling by Retinoic Acid	R-BTA-5362517	0.0074
Pyruvate metabolism	R-BTA-70268	0.0085
Biotin transport and metabolism	R-BTA-196780	0.0088
Energy dependent regulation of mTOR by LKB1-AMPK	R-BTA-380972	0.0107
Metabolism of lipids	R-BTA-556833	0.0110
Gap-filling DNA repair synthesis and ligation in TC-NER	R-BTA-6782210	0.0120
Cytochrome P450 - arranged by substrate type	R-BTA-211897	0.0124
GRB2:SOS provides linkage to MAPK signaling for Integrins	R-BTA-354194	0.0136

^a^ Using Bovine mine database v.1.4

## Discussion

Currently, little is known about MAP’s mechanisms to restrain macrophages from becoming the main reservoir that ensures its multiplication and survival. Macrophages play a central role in mycobacterial pathogenesis. Therefore, macrophages are used as a model for *M. tuberculosis* and *M. bovis* [60–63], and to study MAP [59, 64, 65]. Because some cows are more resistant to paratuberculosis, we hypothesized that macrophages could be used as a sub-system to identified genetic variations associated to JD. Two-pronged issues arise, on one hand, MAP is able to subvert the functions of infected macrophages to establish its protective environment, and on the other hand, the host genetics influences MAP infection success. In a previous preliminary study, we depicted a significant difference in the transcriptome of primary bovine macrophages from JD (+) and (−) cows [57] where macrophages from JD(+) cows can reproduce complex phenotypes observed in tissue, like giant epithelioid cells, foam cells, and granulomatous appearance with high endogenous lipid droplets. In the current study, we used RNA-seq to provide a high-resolution extent of the genetic variations between the two groups, JD (+) and (−) cows. The result allowed the identification of novel genes and pathways involved in MAP infection. This study presents an opportunity to uncover the genetic aspect of both JD (+) and (−) cows using DNA and RNA-seq genotyping methods. In contrast to previous studies, UTR and intronic variants dominated most RNA-seq variants [58, 66]. One could attribute this skewed distribution to the limited knowledge about alternative splicing in bovine species or the absence of methylated cytosine in CpG dinucleotides in exonic regions, reported to bias SNP calling in DNA-based sequencing methods [67]. Moreover, numerically more variant exists in introns than in coding regions since selective pressure plays a role in variant distribution on the genome [68]. Indeed, alternative splicing contributes to producing numerous mature transcripts from the same primary RNA sequence. In other words, the pattern of whole-exome sequencing may exclude some intronic sequences with an essential functional role. Alternative splicing occurs in over 90% of genes [69], limiting the scope of whole-exome sequencing, whereas the RNA-seq method can still uncover the genetic variations from all alternative transcripts. Given the very high number of spliced variants and non-coding RNA in humans [58] and the lack of equivalent knowledge for the bovine species, RNA-seq remains an exciting approach to study the entire transcriptome and to uncover genetic variations from the non-coding and all alternative transcripts.

To our knowledge, this is the first GWAS for susceptibility to paratuberculosis using a dense RNA-seq dataset. The approach combines RNA-seq with a GWAS as it is an effective method for identifying expression quantitative trait loci (eQTL). We compared two cohorts of 22 JD (+) and 28 JD(−) cows. We identified a total of 1,356,248 markers, of which 52,360 variants had a high, low, or moderate impact on the transcriptomic machinery or the coding sequence. Among these, 4810 SNPs located in the vicinity of the alternative splicing sites are believed to exert control on the type of mRNA isoform. While most of these SNPs are more than 1 kb of a transcription start site, other effects such as epistasis or linkage equilibrium make it challenging to identify the SNPs with the main effect. Nonetheless, in this preliminary study, we demonstrate that this approach is a robust way to identify eQTL functional markers associated with paratuberculosis using macrophages as a subsystem.

Though most significant genetic variants were mapped to many genes, some SNPs were localized to specific regulatory regions, whereas others may influence gene expression and related pathways. Interestingly, many enriched pathways were associated with amino acid metabolism roles, energy production, and lipids metabolism. Only four of the top 15 pathways were not directly associated with those categories indicating that the variants linked to the JD susceptibility might impact genes with the host’s metabolism roles. Moreover, two of the genes mapped by highly-significant peak of variants on BTA4 (*PNPLA8 gene)* and BTA11 (*CYP26B1 gene)* are implicated in metabolism pathways. *PNPLA8 gene*, which encodes for the Phospholipase A2 Gamma, is located mainly in the peroxisome and mitochondria. These phospholipases help catalyze the cleavage of fatty acids from the membrane’s phospholipids. It also has a prominent effect on the modulation of energy storage and lipid utilization [70]. *CYP26B1 gene* encodes for the cytochrome P450 Family 26 Subfamily B Member 1 protein. This enzyme catalyzes reactions implicated in the synthesis of cholesterol and other lipids while also acting as a regulator of the retinoic acid level. However, GWAS significant SNPs had a putative impact on *the NRCAM gene* (Table 3), a β-catenin target gene. The *NRCAM* gene is a member of the immunoglobulin superfamily that encodes a cell adhesion molecule with multiple immunoglobulin-like C2-type domains and fibronectin type-III domains. Aberrant β-catenin signaling activation is associated with chronic inflammation and organ fibrosis [71, 72]. The failure to resolve acute inflammation leads to chronic inflammation, tissue remodeling, and fibrosis [73]. Defective β-catenin-*NRCAM* signaling may have a subsequent role in granulomatous formation. This adhesion molecule is downregulated in dendritic cells during inflammation [74]. In our previous study, *NRCAM* was found downregulated in macrophages in response to MAP infection but was not differentially expressed in JD(+) vs. JD(−) macrophages (data not shown). In other studies, the *NRCAM* gene overlapped with QTL found in association with milk composition [75], protein percentage (rs41650658) [76], and milking temperament (rs41587635) [77]. The impact of this gene on production and functional traits for dairy cattle is not known. Besides, the role of *NRCAM* in foam cell and fibrosis phenotypes observed in JD(+) macrophages requires further investigation. The change in fatty acid metabolism and resolution of inflammation through the foam cell and fibrosis phenotypes could be a way by which MAP survive and evade the immune system to establish a persistent infection, leading to JD.

Difficulties in studying JD include the identification of infected animals and the ability to match a non-infected animal in commercial herds. It is accepted that MAP infection’s susceptibility occurs early in life and that disease manifests in four stages: silent, subclinical, clinical, cachexia [1, 10]. Identifying an infected animal depends on the testing frequency and the ease with which the disease can be detected using current laboratory methods. Quantitative PCR was used as is more sensitive than MAP culture [17, 78]. The serological test we used (ELISA IDEXX) is highly specific (96–99%) and its sensitivity is 75% for MAP shedding cows [13] and 99.2% for combined tests [79]. Cows were tested every 6 months during the longitudinal study (3–5 yrs. period) using blood ELISA and fecal PCR methods to define JD status. ELISA and fecal PCR are well recognized phenotypes for the identification of MAP infected cows or cows diagnosed with paratuberculosis disease [44]. The concurrent positive detections (blood and feces) indicate that the 22 cows were JD positive, and no misleading false-positive diagnosis was attributed to JD(+) cows. The JD(−) cows were non-infected herd mates with no trace of MAP in feces and were concomitantly testing ELISA negative. Some non-infected cows might have been exposed but were free of MAP excretion throughout the study. Clinical signs appear 2–7 years after the infection. Because they tested negative every 6 months until they were culled at later age > 7 yrs. old, and since there were no intermittent shedding and absence of MAP-specific antibodies in blood during the longitudinal study (3–5 yrs. period), cows were not JD positive.

The population used in our study is small, owing to the challenge of collecting a substantial amount of blood on cows on commercial herds having received the diagnosis of JD and ready to be culled. Diagnosis and culling of JD animals are ineffective preventive measures. Because of slow progression, the disease is systematically diagnosed too late, while subclinical cows would have excreted MAP sporadically in the environment. As the infection and disease progress, the fecal shedding of MAP increases and contributes to its horizontal transmission. In combination with genetic improvement (innate protection), vaccination (acquired protection) will support eradicating this incurable disease. The Canadian dairy industry is aware of the importance of considering genetic resistance to MAP infection. To this end, molecular markers relevant to JD are needed to improve Holstein breeding programs, the dairy industry’s predominant breed. The importance of defining JD-associated markers having a significant variance effect on the trait is a challenge because of the limited study sample size, high linkage disequilibrium in dairy cattle, and the disease’s low heritability. For instance, some SNPs are linked with paratuberculosis disease and described as risk factors but could not be associated with the disease for two reasons: one reason concerns the relevance of the population tested (different breeds) [49], and the second concerns the functionalities of the SNPs, which have rarely been investigated. Our results suggest that the discovery of genetic variants using the transcriptome is an attractive complementary approach to the traditional DNA chip genotyping method. However, this strategy requires intensive laboratory manipulations (e.g., isolation of blood immune cells and macrophage culture). Alternatively, genotyping by sequencing could also be an exciting approach to discover variants in non-repetitive and single-copy gene-rich regions of the genome [80] that can complement DNA chip genotypes [81]. The macrophage subsystem used in this study allows studying early infection events in a controlled environment. Previous results indicate macrophages have been used as a model to understand the biological and molecular pathways affected during MAP infection [59, 82, 83]. It is also considered a new dimension in the infection model to study host genetics [84]. A strategy to discover functional markers associated with a phenotype is essential for the dairy industry. With the availability of the full bovine genomic sequence, numerous SNPs are available. However, we still have the daunting tasks of predicting the ‘genotype-to-phenotype’ effect, mainly because MAP resistance/susceptibility is lowly heritable and is a complex trait affected by many genes that have not yet been defined.

## Conclusion

In this research, we have identified functional markers associated with JD. The SNP identified using macrophages as the subsystem can potentially explain the establishment of the foam cell and fibrosis phenotypes associated with the granulomatous appearance observed in JD(+) macrophages. The beneficial or unfavorable impact of these eQTLs remains to be investigated before been considered in genetic selection. If confirmed, the dissemination of beneficial allele(s) in the progeny should support the host immune system’s permanent effect. This research’s originality lies in the use of a simplified system that uses macrophages in vitro to control environmental parameters. RNA-seq provided data which, coupled with microarray genotypes, enabled high-resolution genomic analysis to identify new mutations, transcripts, and novel pathways associated with bovine paratuberculosis.

## Methods

### Animal selection and JD diagnosis

Fifteen dairy herds in Canada, located in the province of Quebec, were included in the study. Cows were sampled on commercial farms for the duration of the study. They were owned by dairy producers and were kept on farms beyond the end of sampling. Animals were treated according to local management practices and as recommended by the attending veterinarian. They were not euthanized as part of this study. A confidentiality agreement has been signed with all dairy producers. Cows had to be at least 30 months old at first sampling and paired blood and fecal samples (collected at the same time) were analyzed twice per year (every 5 to 7 months) as described [17]. They were sampled during a three-to-five-year period to exclude animals still in the silent period. JD(+) cows or JD(−) were selected and some of them were also part of a companion project [17, 59]. Cows that presented concordant serological and fecal culture or qPCR statuses, either positive or negative, were retained. As previously described [17], cows were considered to be shedders if the qPCR threshold cycle (Ct) was 35 or less, and non-shedders if Ct was 45. Regarding the ELISA testing kit (IDEXX Laboratories, Markham, ON, Canada), the threshold of 45 was used (S/P higher than 45% according to manufacturer). All samples with intermediate qPCR results (> 35) or serological results (> 5 and < 45) were considered to be of uncertain status and were excluded from the study. ELISA and PCR analyses were performed in a single laboratory with the same test kits throughout the experiment as described [17]. The culture of MAP was performed by the Quebec Animal Epidemiological Surveillance Laboratory (Saint-Hyacinthe, Québec, Canada), as described in [85]. Selected animals were confirmed JD(+) or JD(−) following concordant results for the two consecutive sampling periods. Fifty cows were selected including 6 JD(+) and 6 JD(−) cows analyzed previously [59], and totalized 28 JD(−) cows and 22 JD(+) confirmed using both tests. Negative cows for JD were 6.4 ± 1.5 years, and JD(+) cows were 5.1 ± 1.8 years.

### Differentiation of monocyte-derived macrophages and in vitro MAP infection

For monocyte isolation, blood (700–750 mL) was collected in citrate–phosphate–dextrose–adenine anticoagulant transfusion bags (Animal Blood Resources International, Dixon, CA, USA). Monocytes were isolated peripheral blood mononuclear cells by adherence [86] with minor modifications as described [59].

To differentiate the monocytes into macrophages, cultures were maintained in a humid atmosphere at 39 °C with 5% CO_2_ for a period varying from a week to 10 days. Medium was refreshed every 3 days. The purity of the culture was analyzed by flow cytometry on the 3-laser FACSCanto II flow cytometer (BD Biosciences) using a maker of the macrophage population (anti-CD68 antibody). The anti-CD68 antibody is specific to a protein present on the lysosomal membrane of the macrophages [87]. The procedure includes a fixation and permeabilisation treatments performed using Cytofix/cytoperm kit (BD biosciences, Mississauga, Ontario, Canada). Immuno-labelling included the mouse primary anti-CD68 antibody (M071801–5; Agilent) following by the second anti-Mouse PE labelled IgG1 (P-21129; ThermoFisher, Waltham, MA, USA). The cells displayed macrophage morphology after 7–10 days. One day prior the MAP infection experiments, the culture was rinsed with RMPI 1640 medium (Wisent, Saint-Bruno, Québec, Canada) and incubated without the antibiotics/antimycotics with complete media including 10% bovine heterologous serum (heat-inactivated) collected from tested JD- cows of the local herd (Research and Development Center of Sherbrooke). A mix of GlutaMax (Life Technologies, Burlington, Ontario, Canada) and L-glutamine (Wisent) was added to the media. The culture of MAP was performed in Middlebrook 7H9 liquid media supplemented with mycobactin J as described previously [59]. The in vitro experiments were performed at 39 °C at a multiplicity of MAP infection of 10 bacteria per macrophage. Briefly, the fresh culture of the clinical MAP strain was vortexed, washed, and resuspended in PBS (pH 7.4). The suspension is then passed through a 26G needle to disperse clumps as described [59]. After 15 min of decantation, the concentration of the upper 5 mL layer was measured by absorbance at 600 nm as described elsewhere [88–92]. Macrophage infections were performed, as described elsewhere [59, 93, 94]. The MAP infection flasks were collected at 1 h, 4 h, 8 h, and 24 h. The experiment also included two uninfected control flasks incubated for 4 h and 24 h. Prior to harvest using the RLT buffer (Qiagen, Toronto, Ontario, Canada), the macrophages were washed twice with PBS. Cells were harvested by adding 1.8 mL of lysis buffer to the culture flask. Harvested cells were stored at − 80 °C until RNA extractions were performed.

### RNA extraction, libraries and sequencing

Twelve cows were from a companion project [59]. The RNeasy kit (Qiagen) was used to extract RNA from those MDM. The DNase treatment was performed on-column according to the manufacturer’s recommendations. MDM was prepared from additional 38 animals. A different protocol was used to extract the RNA because the classic RNeasy protocol does not extract miRNA. To retain miRNA along with total RNA, the lysed MDM samples in the buffer RLT were treated using a phenol–chloroform extraction method. Both miRNA and total RNA from the lysed MDM samples were treated using TRIzol LS (Thermo Fisher) and chloroform separation. Both miRNA and total RNA part of the aqueous phase were then extracted using the miRNeasy kit according to the manufacturer’s recommendations. Quantification of the RNA concentration from the six RNA samples per animal (two controls and four infection time points) was performed using a NanoDrop (Thermo Fisher). Quality of the RNA extracts was evaluated using the Bioanalyzer RNA 6000 kit (Agilent Technologies, Santa Clara, CA, USA).

The ribosomal RNA was removed using the Ribo-Zero Gold kit (Illumina, Victoria, British Columbia, Canada). To construct each cDNA library, 250 ng of total RNA was processed using the Illumina TruSeq Stranded Total mRNA Sample Preparation kit (Illumina). In the previous study, one library was generated for each infection time points for the 12 cows [59]. For each additional 38 cows, equal quantity of total RNA from each time points were pooled. For each cow, all genes expressed in the unchallenged macrophages (CTL) were pooled to the transcripts harvested at each infection time points (1 h, 4 h, 8 h, and 24 h). Therefore, all genes that are expressed in the resting macrophages and the genes potentially upregulated during the infection are represented in each library, i.e. for each cow. RNA libraries were constructed as described above and submitted for sequencing to The Centre for Applied Genomics, The Hospital for Sick Children (Toronto, Canada) for quality control prior sequencing. Pool of RNA libraries was quantified by qPCR using the Kapa Library Quantification Illumina/ABI Prism Kit protocol (KAPA Biosystems), cluster on a cBot instrument and paired-end sequenced on 2 lanes of a High Throughput Run Mode flowcell with the V4 sequencing chemistry on an Illumina HiSeq 2500 platform running HCS software v2.2.68 following Illumina’s recommended protocol to generate paired-end reads of 126-bases in length.

### RNA-seq-derived SNP calling and DNA genotyping using bovine SNP50 BeadChip

For each 12 cows previously analyzed [59], RNA-seq reads of the 6 distinct libraries (4 infection time points and 2 controls) were pooled to produce a FASTQ file containing roughly 360 million paired-end (PE) reads per cow. The FASTQ sequence files from the 38 additional cows contained a minimum of 60 million PE reads each. RNA-seq samples were processed in accordance with the Best Practices workflow for variant calling on RNA-seq data up to the variant calling step, after which we switched to the Joint Variant workflow (GVCF mode), a strategy that we validated previously [95]. Briefly, reads were firstly mapped against the UMD 3.1.1/bosTau8 assembly using the STAR aligner (version 2.4.0j, [96]). Next, duplicated reads were marked with Picard tools. The Split’N’Trim and indel realigments steps were performed (Genome Analysis Toolkit, GATK, version 3.3–0-g37228af [97];). The HaplotypeCaller algorithm was used to call variants (Supplementary file 1). With all the gVCFs files derived from the above mentioned step, we performed a joint genotyping analysis for all samples and produced a final VCF file (Supplementary file 2).

All animals were genotyped with the commercial DNA chip. Blood samples (EDTA vials) were collected at the first sampling with the registered animal ID. Extraction of the genomic DNA was performed using the Wizard Genomic DNA Purification Kit (Promega, Madison, WI) following the manufacturer’s instructions. Concentration was evaluated using a NanoDrop ND-1000 spectrophotometer (Thermo Scientific, Wilmington, DE). Genotyping was performed using the Illumina BovineSNP50 BeadChip (Zoetis, Kalamazoo, MI, USA) by Zoetis (Sainte-Anne-de-Bellevue, Qc, Canada). Using a custom Perl script [81], a VCF file was generated from the Illumina ‘A/B’ allele genotypes, which contains 67,740 SNPs. This script removed SNPs not consistent with the nomenclature of ‘Allele A’ and ‘Allele B’ according to the Illumina genotyping system. The script remove also duplicate SNPs and SNPs that do not perfectly aligned (BLASTN) to the reference sequences of the mitochondrial genome, the bovine autosomes, or the X chromosome (UMD 3.1.1/bosTau8). Dataset of 50,539 was obtained SNPs from the BovineSNP50 BeadChip. Imputation to the BovineHD density was performed with FImpute v2.2 (Sargolzaei et al., 2014) using a large reference panel which we had access through the Center of Genetic Improvement of Livestock (CGIL) at Guelph University, Ontario, Canada.

### Variant filtering, and annotation

RNA-seq-derived SNP and BovineSNP data were merged and imputed using Minimac3 (version 2.0.1) [98] software tools. SVS v8.7.1 (Golden Helix) and the BCFtools (Li et al. 2009) were used to obtain high quality variants. RNA-Seq genotypes supported by read depth < 4 reads or genotype quality score < 20 were filtered out. Additional variants were eliminated from downstream analysis: those with Minor allele frequency (MAF) < 0.05, those with a Call rate < 0.2, those harboring two or more alternative alleles, and those that were not in Hardy-Weinberg equilibrium (*P* < 0.001). Known variants were annotated with a bovine reference VCF file (SNPdb-version 150) downloaded from the NCBI ftp site [99]. The final constructed dataset used for GWAS analysis is summarized in Supplementary Figure 1.The GWAS statistical analysis was performed using the SVS software by doing a mixed linear model analysis (MLM) with identity by descent estimation and the first 20 principal components accounting for heterogeneous variances and cryptic relatedness. All parameter of the analysis used in the SVS software are consigned in the Supplementary file 3.

### Functional analysis (pathways and networks)

The software SnpEff v.4.3 t [100] was used to predict the functional or biologically interpretable pathways and networks associated with significant variants. The final aim of this study is to characterize the variants that may be of special interest for the dairy industry. Considering that, we used SnpEff to detect variants associated with candidate genes related to JD. Using BovineMINE v1.4, functional analysis of the pathways and networks was performed [101].

## Supplementary Information

**Additional file 1: Supplementary Figure 1.** Schematic representation of the approach used to generate SNPs for GWAS analysis. The Bovine 50 K DNAchip data were imputed to the BovineHD using a reference panel of 3300 HD animals. After filtering to generate high-confidence set of variants, including RNA-seq, and DNA chip variants, the matched RNA and DNA samples enable verification of RNA SNP calls because they can be compared to the variant present in the respective dataset. Missing variants were imputated using Minimac3.

**Additional file 2: Supplementary Figure 2.** Principal component analysis (PCA) of the 50 datasets of DNA variant. The PCA plots of each cow from both JD groups were drawn using the stats R package. All R analyses and graphs were processed in RStudio v0.99.467.

**Additional file 3: Supplementary Figure 3.** Visualization of the GWAS results using Quantile-Quantile (Q-Q) plot. GWAS examined hundreds of thousands of DNA variants in 50 cows, testing their statistical association with discrete outcomes, JD case-control study. Q-Q plots display the observed association *P*-value for all SNPs on the y-axis versus the expected uniform distribution of *P*-values under the null hypothesis of no association on the x-axis. Strongly associated SNPs will deviate from the diagonal at the upper-right end of the plot, while systematic deviation from the diagonal may indicate problems with the data, such as population stratification or cryptic relatedness.

**Additional file 4: Supplementary file 1.**

**Additional file 5: Supplementary file 2.**

**Additional file 6: Supplementary file 3.**

## Data Availability

All sequencing data are available in the NCBI Gene Expression Omnibus (GEO) database under the accession number GSE98363 (https://www.ncbi.nlm.nih.gov/geo/query/acc.cgi?acc=GSE98363). The reference sequences of the mitochondrial genome, the bovine autosomes and the X chromosome are from the UMD 3.1.1/bosTau8 assembly and are publically available at NCBI under the accession number GCF_000003055.6 (https://www.ncbi.nlm.nih.gov/assembly/GCF_000003055.6) or alternatively at the Bovine Genome Database (http://128.206.12.216/drupal/sites/bovinegenome.org/files/data/umd3.1/UMD3.1_chromosomes.fa.gz).

## Abbreviations

eQTL: Expression quantitative trait loci
GWAS: Genome-wide association study
HWE: Hardy-Weinberg equilibrium
JD: Johne’s disease
MAF: Minor allele frequency
MAP: *Mycobacterium avium subsp. paratuberculosis*
PCR: Polymerase chain reaction
RNA-Seq: RNA sequencing
SNP: Single-nucleotide polymorphism
