# Supplementary material for: Genome-wide association analysis identified both RNA-seq and DNA variants associated to paratuberculosis in Canadian Holstein cattle ‘in vitro’ experimentally infected macrophages
Source: BMC Genomics. 2021 Mar 7;22:162. doi: 10.1186/s12864-021-07487-4 (PMC7938594; doi:10.1186/s12864-021-07487-4)
Supplement: Supplementary file 1 — Additional file 1: Supplementary Figure 1. Schematic representation of the approach used to generate SNPs for GWAS analysis. The Bovine 50 K DNAchip data were imputed to the BovineHD using a reference panel of 3300 HD animals. After filtering to generate high-confidence set of variants, including RNA-seq, and DNA chip variants, the matched RNA and DNA samples enable verification of RNA SNP calls because they can be compared to the variant present in the respective dataset. Missing variants were imputated using Minimac3. [file 12864_2021_7487_MOESM1_ESM.pdf]

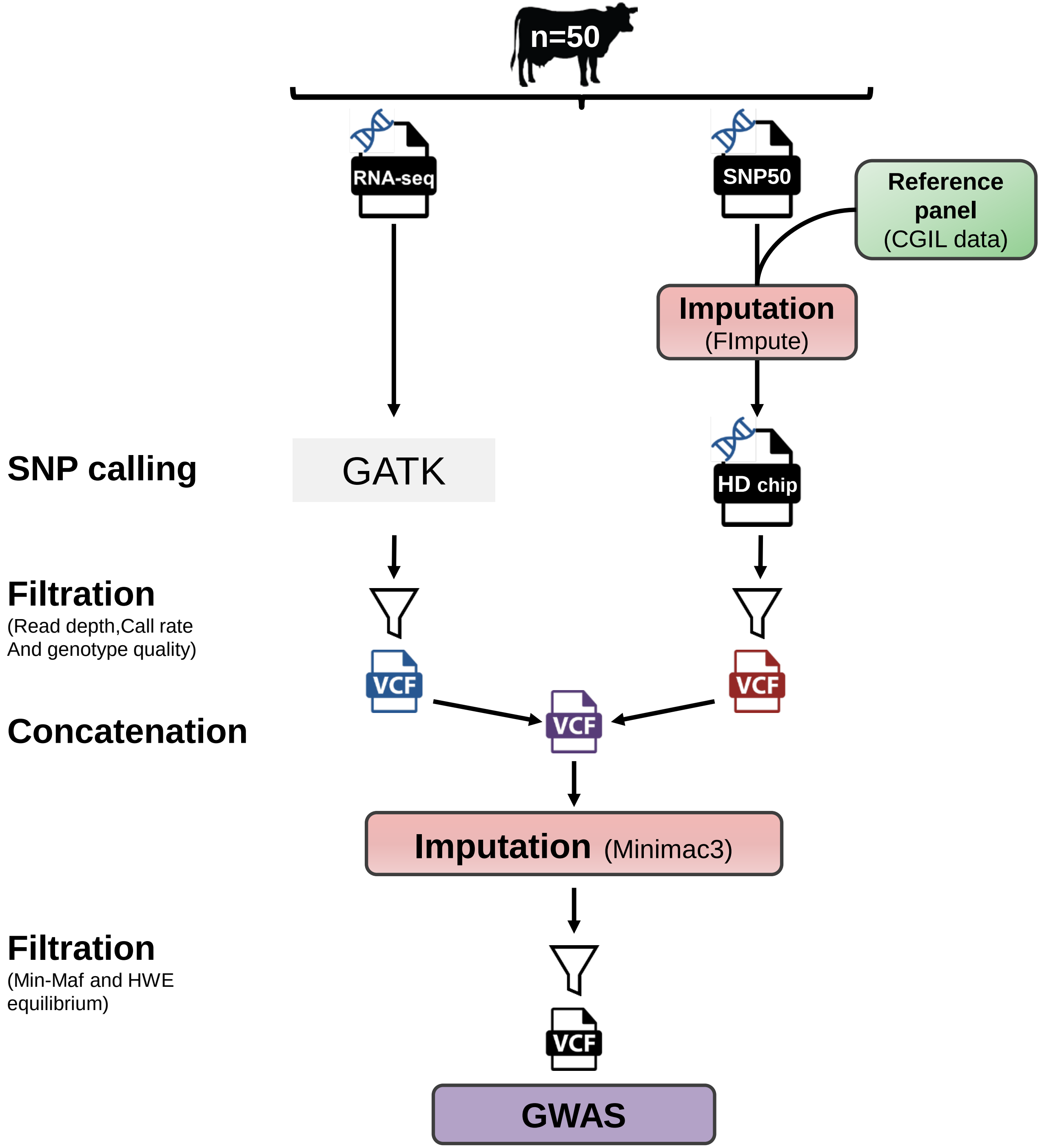

n=50
RNA-seq
SNP50
Reference panel (CGIL data)
Imputation (FImpute)
SNP calling
GATK
HD chip
Filtration
(Read depth,Call rate And genotype quality)
VCF
VCF
Concatenation
VCF
Imputation (Minimac3)
Filtration
(Min-Maf and HWE equilibrium)
VCF
GWAS
