# Supplementary figures and images for: Genome-wide association analysis identified both RNA-seq and DNA variants associated to paratuberculosis in Canadian Holstein cattle ‘in vitro’ experimentally infected macrophages
Source: BMC Genomics. 2021 Mar 7;22:162. doi: 10.1186/s12864-021-07487-4 (PMC7938594; doi:10.1186/s12864-021-07487-4)

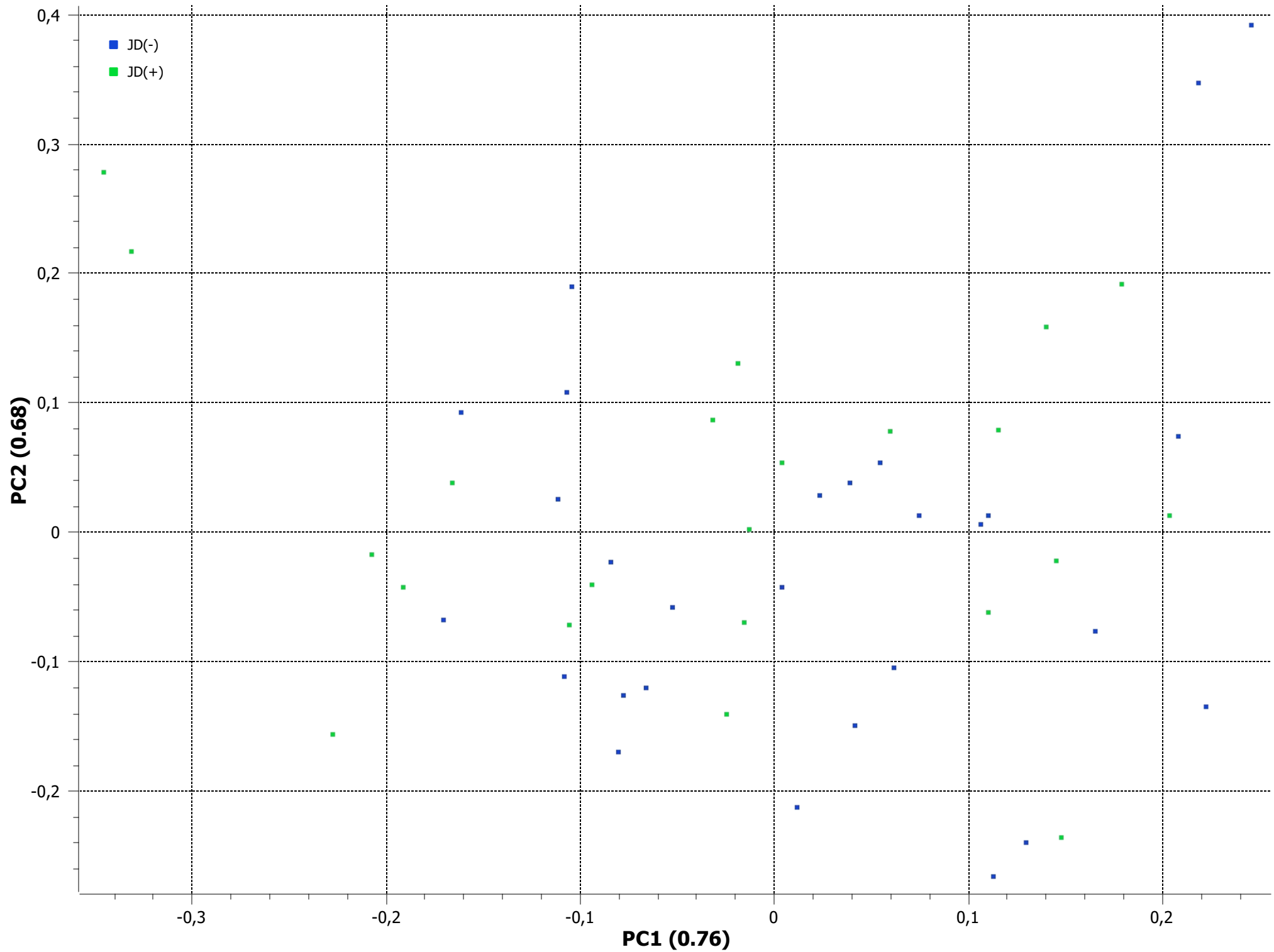

Supplement: Supplementary file 2 — Additional file 2: Supplementary Figure 2. Principal component analysis (PCA) of the 50 datasets of DNA variant. The PCA plots of each cow from both JD groups were drawn using the stats R package. All R analyses and graphs were processed in RStudio v0.99.467. [file 12864_2021_7487_MOESM2_ESM.pdf]

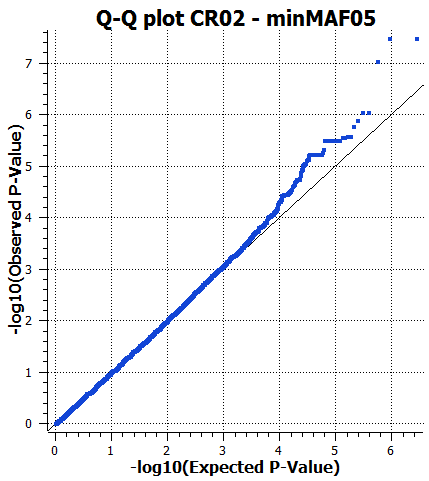

Supplement: Supplementary file 3 — Additional file 3: Supplementary Figure 3. Visualization of the GWAS results using Quantile-Quantile (Q-Q) plot. GWAS examined hundreds of thousands of DNA variants in 50 cows, testing their statistical association with discrete outcomes, JD case-control study. Q-Q plots display the observed association P-value for all SNPs on the y-axis versus the expected uniform distribution of P-values under the null hypothesis of no association on the x-axis. Strongly associated SNPs will deviate from the diagonal at the upper-right end of the plot, while systematic deviation from the diagonal may indicate problems with the data, such as population stratification or cryptic relatedness. [file 12864_2021_7487_MOESM3_ESM.png]
